# Etiology of Papilledema in Patients in the Eye Clinic Setting

**DOI:** 10.1001/jamanetworkopen.2020.6625

**Published:** 2020-06-02

**Authors:** Olivia M. Crum, Khin P. Kilgore, Rishi Sharma, Michael S. Lee, Matthew R. Spiegel, Collin M. McClelland, M. Tariq Bhatti, John J. Chen

**Affiliations:** 1 Alix School of Medicine, Mayo Clinic, Rochester, Minnesota; 2 Department of Ophthalmology, Mayo Clinic College of Medicine, Rochester, Minnesota; 3 University of Minnesota College of Biological Sciences, Minneapolis; 4 Department of Ophthalmology and Visual Neurosciences, University of Minnesota, Minneapolis; 5 Department of Health Sciences Research, Mayo Clinic, Jacksonville, Floriada; 6 Department of Neurology, Mayo Clinic College of Medicine, Rochester, Minnesota

## Abstract

**Importance:**

The study of health conditions associated with papilledema will augment the clinical judgment of eye care professionals treating patients with optic disc edema in determining the urgency of additional evaluation and counseling patients accordingly.

**Objectives:**

To determine the incidence, demographic characteristics, and etiologies of papilledema based on a unique records–linkage research platform; and to describe the demographic and clinical differences between patients with idiopathic intracranial hypertension (IIH) and other causes of papilledema.

**Design, Setting, and Participants:**

Retrospective population-based cross-sectional study of patients treated for papilledema at outpatient eye clinics in Olmsted County, Minnesota, using the Rochester Epidemiology Project. Data were collected from January 1990 to December 2014 and analyzed from September 2018 to April 2019.

**Main Outcomes and Measures:**

Etiologies of papilledema, body mass index, incidence of headache, or localizing neurologic signs.

**Results:**

Eighty-six patients were diagnosed with papilledema during the 24-year period, providing an age- and sex-adjusted incidence of 2.5 individuals per 100 000 per year; 68 patients (79%) were women, 73 (85%) were white patients, and the median (range) age was 27.7 (6.2-64.2) years. Nineteen patients (22%) presented with a previously diagnosed attributable cause (eg, trauma or intracranial tumor). Among patients presenting with papilledema without a previously diagnosed attributable cause, 58 patients (87%) had IIH, and 9 patients (13%) were found to have a secondary cause of raised intracranial pressure, such as intracranial tumor, cerebral venous sinus thrombosis, or granulomatous meningitis. Patients with IIH had a higher median (range) body mass index (37.5 [20.4-55.7] vs 27.4 [16.6-40.1]; *P* = .003) and headache prevalence (54 of 58 patients [93%] vs 6 of 9 patients [67%]; *P* = .004) than patients with other causes of papilledema. Of 9 patients with papilledema but no IIH, 2 (22%) had localizing neurologic signs, such as gait abnormalities, hearing loss, focal weakness or numbness, visual field defects, or aphasia. Among 42 patients with demographic characteristics typically associated with IIH (female sex, with obesity, aged 15 to 45 years, and absent localizing neurologic signs or symptoms), 40 (95%) had papilledema that was associated with IIH. Conversely, among the 19 patients without these demographic characteristics, 7 (37%) had an alternative cause.

**Conclusions and Relevance:**

In this study, most patients who presented to the eye clinic with papilledema without a previously known cause were found to have IIH. These patients were more likely to present with headaches and had statistically higher body mass index. Clinicians should take these findings into account when determining the pretest probability of a patient having IIH or an alternative cause of papilledema.

## Key Points

**Question:**

What is the likely etiology of papilledema among patients presenting to an eye clinic without a previously known cause?

**Findings:**

In this population-based cross-sectional study including 86 patients diagnosed with papilledema, most patients (87%) were found to have idiopathic intracranial hypertension, while 13% presented with potentially life-threatening conditions, including intracranial tumor, cerebral venous sinus thrombosis, and granulomatous meningitis.

**Meaning:**

All patients presenting with papilledema require neuroimaging; however, based on the results of this study, those with the typical demographic characteristics of idiopathic intracranial hypertension and lack of other neurologic symptoms may not need urgent neuroimaging, as long as the papilledema is not severe enough to threaten vision.

## Teaser

This cross-sectional study examines the relative frequency of etiologies of papilledema and describes the clinical and demographic differences between patients with idiopathic intracranial hypertension and other causes of papilledema.

## Introduction

Papilledema is an important finding that can herald a serious neurologic condition. However, to our knowledge, there has not been a population-based study of its incidence and etiologies in an outpatient eye clinic setting. Specifically, if a patient presents to an eye care professional with optic disc edema, what is the likelihood that it is papilledema caused by a life-threatening condition? While many cases of bilateral papilledema are caused by idiopathic intracranial hypertension (IIH), some will be caused by other etiologies, such as obstructive hydrocephalus, cerebral venous sinus thrombosis, and intracranial masses, among others. This study examined medical records from the Rochester Epidemiology Project to determine the relative frequency of etiologies of papilledema in patients visiting outpatient eye clinics.

## Methods

In this population-based, cross-sectional incidence study, medical records were identified using the Rochester Epidemiology Project database, a multicenter medical records–linkage system for persons residing in Olmsted County, Minnesota, that was established in 1966.^1,2^ This medical records–linkage system includes medical data from multiple institutions, encompassing almost all sources of medical care used by the Olmsted County population. Data shared across institutions within a geographically defined population allow for indispensable population-based research.^1,2,3^

This study was approved by the institutional review board of the Olmsted Medical Center, Minnesota. The Mayo Clinic institutional review board waived the need for informed consent for this study because this was a retrospective medical record review with minimal risk. This study adheres to the tenets of the Declaration of Helsinki.^4^ All participants provided written consent to the use of their information for research. This study followed the Strengthening the Reporting of Observational Studies in Epidemiology (STROBE) reporting guideline.

The medical records of all Olmsted County residents with newly diagnosed papilledema from January 1990 through December 2014 were identified using the Rochester Epidemiology Project database by searching for IIH, intracranial hypertension, pseudotumor cerebri, or papilledema diagnosis codes. The medical records of the 427 patients initially identified were individually reviewed to confirm the diagnosis of papilledema and determine residency in Olmsted County at the time of diagnosis. All borderline cases were reviewed by a neuro-ophthalmologist (J.J.C.) to confirm or refute the diagnosis of papilledema.

Patients who were referred to our institution but were not residents of Olmsted County were excluded. Patients who did not have disc edema documented in an outpatient eye clinic were also excluded from the study to ensure findings are most relevant for eye care professionals encountering a new diagnosis of disc edema in the clinic. For this study, the term *eye clinic* refers to both ophthalmology and optometry clinics. Optic disc edema and true papilledema were carefully distinguished; patients with optic disc edema without increased intracranial pressure, such as in optic neuritis, pseudopapilledema, and ischemic optic neuropathy, were excluded.

For each patient who met the inclusion and exclusion criteria, outpatient records were reviewed for possible conditions related to intracranial pressure, including IIH, intracranial tumor, head trauma, intracranial hemorrhage or ischemia, meningitis, encephalitis, venous sinus thrombosis, neurosarcoidosis, hydrocephalus, aqueductal stenosis, and others. Patients were classified as having IIH if they met the Modified Dandy criteria.^5,6^

Data on sex, race, age at diagnosis, body mass index (BMI; calculated as weight in kilograms divided by height in meters squared), and symptoms were obtained. Patient race and ethnicity were self-reported from options defined by the institution. Race was collected to demonstrate the probable racial homogeneity of the study population. Although all the patients in this study had papilledema as a new finding, those with a preexisting condition that accounted for the papilledema, such as trauma, were identified. Localizing neurologic signs or symptoms, fever, or altered mental status were also documented. As per the Modified Dandy criteria for IIH,^5,7^ sixth nerve palsy was not considered a localizing neurologic finding.

### Statistical Analysis

The overall incidence of papilledema was estimated using the total number of cases divided by the population of Olmsted County in that period. Overall populations of Olmsted County were estimated using the census results as well as an interpolation of population between the census years. Incidence rates were age- and sex-adjusted to the 2010 US white population, indicating the expected number of new cases per 100 000 individuals per year if our study population had the same age and sex distribution as the white population did in 2010. Comparisons between groups for any categorical factors were completed using the Fisher exact test. Continuous variables were compared using Wilcoxon rank sum tests. Statistical significance was set at *P* < .05, and all tests were 2-tailed. Analysis was completed using R version 3.6.1 (R Project for Statistical Computing) and SAS version 9.4 (SAS Institute).

## Results

Eighty-six patients were diagnosed with papilledema in an outpatient eye clinic setting during the 24-year study period, providing an age- and sex-adjusted incidence of 2.5 individuals per 100 000 per year; 68 patients (79%) were women, 73 (85%) were white patients, and the median (range) age was 27.7 (6.2-64.2) years.

Of the 86 patients newly diagnosed with papilledema, 19 (22%) had a previously diagnosed attributable cause of papilledema. Among patients presenting with papilledema without a previously known cause, 58 (67%) were diagnosed with IIH, and 9 (10%) were found to have a secondary cause of raised intracranial pressure. The most common causes of papilledema without IIH were intracranial tumor, intracranial hemorrhage, and cerebral venous sinus thrombosis (Table 1).

**Table 1.** Etiologies of Papilledema

Characteristic	Patients presenting with papilledema, No. (%) (N = 86)							
	Previously diagnosed				Undiagnosed at time of papilledema			
	Intracranial tumor (n = 10)	Intracranial hemorrhage (n = 6)	Venous sinus thrombosis (n = 2)	Neurosarcoidosis (n = 1)	Idiopathic intracranial hypertension (n = 58)	Intracranial tumor (n = 4)	Venous sinus thrombosis (n = 4)	Granulomatous meningitis (n = 1)
Women	5 (50)	1 (17)	2 (100)	1 (100)	53 (91)	2 (50)	3 (75)	1 (100)
Age, median (range), y	38.2 (7.9-58.0)	45.7 (15.2-64.2)	26.0 (24.1-27.9)	41.6 (NA)	27.3 (11.8-48.7)	15.0 (7.0-41.9)	28.4 (6.2-48.5)	26.6 (NA)
BMI, median (range)	23.3 (14.2-38.8)	27.6 (16.7-46.9)	40.3 (39.6-41.0)	25.6 (NA)	37.5 (20.4-55.7)^a^	25.4 (16.7-40.1)	29.8 (16.6-35.1)	33.0 (NA)
Headaches	6 (60)	4 (67)	2 (100)	1 (100)	54 (93)	3 (75)	2 (50)	1 (100)
Focal neurologic deficits	4 (40)	0	1 (50)	0	0	2 (50)	0	0

Abbreviation: BMI, body mass index (calculated as weight in kilograms divided by height in meters squared); NA, not applicable.

^a^ Data were not available for 11 patients.

### Comparison of Patients With IIH vs Patients With Papilledema From Other Causes

Compared with the 28 patients with papilledema from other causes of intracranial hypertension, the 58 patients with IIH were found more likely to be women (53 [91%] vs 15 [54%]; *P* < .001), have a higher median (range) BMI (37.5 [20.4-55.7] vs 26.9 [14.2-46.9]; *P* < .001), and have a headache on presentation (54 [93%] vs 19 [68%]; *P* = .004). No patients with IIH had localizing neurologic symptoms, while 7 patients (25%) with papilledema from non-IIH etiologies demonstrated them.

As per the Modified Dandy criteria for IIH, sixth nerve palsy was not considered a localizing neurologic finding. Within the IIH population, 12 patients (21%) experienced a sixth nerve palsy, while 9 cases (32%) were documented in the non-IIH population (*P* = .37). In the non-IIH population, 1 patient had skew deviation,^8^ which is an acquired vertical misalignment of the eyes indicating an asymmetric disruption of supranuclear input from the otolithic organs.

### Comparison of Patients With IIH vs Without IIH in Cohort With Papilledema Undiagnosed at Time of Presentation

After excluding the 19 patients who presented to the eye clinic with a previously known cause of papilledema, 67 patients presented with an unknown etiology. Among these patients, 58 (87%) were diagnosed with IIH and 9 (13%) were ultimately found to have an intracranial pathology causing increased intracranial pressure (Table 1).

Two of the 9 patients without IIH (22%) had localizing neurologic deficits in addition to papilledema, which were found to be from intracranial tumors (Table 2). The median (range) BMI of the 9 patients with non-IIH etiologies (27.4 [16.6-40.1]) was significantly lower than that of patients with IIH (37.5 [20.4-55.7]) (*P* = .003). Two-thirds of patients without IIH etiologies presented with headaches (67%; 95% CI, 30%-93%), compared with 93% (95% CI, 83%-98%) of patients with IIH (*P* = .046). There were no significant differences in visual acuity upon presentation between patients with and without IIH.

**Table 2.** Characteristics of Patients With Papilledema Undiagnosed at Time of Presentation

Characteristic	Patients with undiagnosed papilledema, No. (% [95% CI])			*P* value
	IIH (n = 58)	Other (n = 9)	Total (n = 67)	
Age, y				
15-45	50 (86 [75-94])	5 (56 [21-86])	55 (82 [71-90])	.047
Median (range)	27.3 (11.8-48.7)	26.0 (6.2-48.5)	27.0 (6.2-48.7)	.35
Women	53 (91 [81-97])	6 (67 [30-93])	59 (88 [78-95])	.07
Race				
White	51 (88 [77-95])	9 (100 [66-100])	60 (90 [80-96])	.99
African American	1 (2 [0-9])	0	1 (1 [0-8])	
Other	4 (7 [2-17])	0	4 (6 [2-15])	
Unknown	2 (3 [0-12])	0	2 (3 [0-10])	
BMI^a^				
>30^b^	44 (85 [72-93])	4 (44 [14-79])	48 (79 [66-88])	.007
Median (range)	37.5 (20.4-55.7)	27.4 (16.6-40.1)	35.5 (16.6-55.7)	.003
Headaches	54 (93 [83-98])	6 (67 [30-93])	60 (90 [80-96])	.046
Localizing neurologic symptoms	0	2 (22 [3-60])	2 (3 [0-10])	.02
Visual acuity, median (range), Snellen fraction				
Right eye	20/20 (20/20-20/200)	20/20 (20/15-20/1000)	20/20 (20/15-20/1000)	.96
Left eye	20/20 (20/15-20/4000)	20/20 (20/15-20/60)	20/20 (20/15-20/4000)	.27

Abbreviations: BMI, body mass index (calculated as weight in kilograms divided by height in meters squared); IIH, idiopathic intracranial hypertension.

^a^ BMI was not available in 6 patients.

^b^ BMI >30 was the predefined value for patients with obesity.

Of the 9 patients without IIH, 2 (22%; 95% CI, 3%-60%; *P* = .02) had demographic characteristics typically associated with IIH (female sex, with obesity, aged 15 to 45 years,^9^ and absent localizing neurologic signs or symptoms), constituting 3% of patients presenting with papilledema of unknown cause. In comparison, 40 of 52 patients (77%) with IIH had these characteristics (*P* = .003). (Six patients missing BMI data or with an unknown obesity status were excluded.) Among patients with demographic characteristics usually associated with IIH, 40 of 42 cases of papilledema (95%) resulted from IIH. Conversely, among patients without these demographic characteristics, 7 of 19 patients (37%) had an alternative cause.

## Discussion

Diagnosis of papilledema generates immediate concern for a life-threatening etiology, such as intracranial mass, obstructive hydrocephalus, meningitis, or cerebral venous sinus thrombosis (CVST). However, our study found that most patients with papilledema have IIH as the underlying etiology, which is a diagnosis of exclusion often occurring in women of childbearing age with obesity. IIH is non–life-threatening^6^ and usually requires a less urgent evaluation unless there is severe papilledema with eminent risk of vision loss. In comparison, other etiologies of papilledema, such as intracranial tumor or CVST, often need rapid workup, diagnosis, and treatment. Knowing the probability of a critical diagnosis aids in the physician’s counseling of the patient and can help determine the urgency of neuroimaging^10^ with subsequent lumbar puncture, as appropriate.

To help guide the evaluating clinician, statistics on demographic characteristics and symptoms have been reported in the literature as evidence for or against a diagnosis of IIH. Among patients presenting with papilledema of unknown cause, our study found that 87% had papilledema related to IIH, while only 13% were from a secondary cause. Among secondary etiologies, only 2 patients (22%) met the IIH demographic characteristics and had no focal neurologic symptoms. Patients with IIH had a significantly higher BMI than those with a secondary cause of raised intracranial pressure, consistent with the findings of Blanch et al.^11^ Our results also showed a 95% chance of papilledema being attributable to IIH if the patient’s demographic characteristics were in line with IIH. Conversely, when papilledema was present among patients not meeting IIH demographic characteristics, 37% of patients were found to have a cause other than IIH. We also found that patients with IIH presented more frequently with headaches, supporting prior studies demonstrating that IIH often presents with substantial headache-related disability over the preceding month.^12,13^

As per the Modified Dandy criteria for IIH, sixth nerve palsy was not considered a localizing neurologic finding. This was seen in roughly equal percentages within our patients with IIH and non-IIH causes of papilledema and is therefore not helpful in distinguishing causes of papilledema. Because of the sixth nerve’s long intracranial course before exiting the skull and its dural tethering, it is vulnerable to intracranial pressure shifts,^14^ regardless of etiology.

The non-IIH etiologies of papilledema seen in our study included intracranial tumor, hemorrhage, meningitis, neurosarcoidosis, and CVST.^15^ These diseases can all be life-threatening and often require urgent, targeted treatment. Among the 4 patients presenting with papilledema secondary to an undiagnosed tumor, 2 had focal neurologic deficits; 1 had headache, sixth nerve palsy, sleepiness, vomiting; and 1 had no symptoms at presentation. Other studies have shown that isolated headache is an uncommon presentation of a brain tumor.^16^

While our patients with CVST had symptoms isolated to raised intracranial pressure, CVST can also cause venous infarctions, severe neurologic deficits, and seizures.^17^ A diagnosis of CVST is important to make to minimize the risk of these more severe central nervous system complications through the initiation of anticoagulation.^18^

All patients presenting with papilledema require neuroimaging. However, based on our results, those with typical IIH demographic characteristics and lack of other neurologic symptoms may not need neuroimaging as urgently, as long as the papilledema is not severe and vision threatening.

### Limitations

This study has limitations, including the retrospective design, the racial homogeneity of the study population, and the small sample size of the non-IIH subgroup. Given the retrospective nature of this investigation, some patients had poor follow-up or incomplete medical records. With 90% of the study population identifying themselves as white patients, it may be difficult to extrapolate these results to other nonwhite populations. Lastly, the study had a small sample size within the non-IIH, unanticipated papilledema subgroup, and therefore was underpowered to evaluate some associations, including differences between men and women. Despite these limitations, we believe our study is unique in analyzing a population-based cohort for etiologies of papilledema in the outpatient setting. This study provides the relative frequency of etiologies of papilledema and the accompanying signs and symptoms, which can be used to counsel patients as the workup of the papilledema is initiated.

## Conclusions

This study found that most papilledema found in the outpatient eye clinic was due to IIH. These patients were more likely to present with elevated BMI and headaches. This study provides beneficial information for the eye care professional faced with an unexpected finding of papilledema.
